# Dietetic Fads and Proteinophoria

**Published:** 1913-02

**Authors:** 


Dietetic Fads and Proteinophoria.—G. Frank Lydstrom of Chicago, *Dietetic and Hyg. Gaz.,* November, 1912, warns against carrying fear of meat too far. He claims that our ancestors were meat-eaters and that man was evolved as a carnivore. We agree with the general warning and with the statement that our ancestors were largely meat-eaters, but incline to the theory that primitive man was mainly nuctivorous and graminivorous.

